# Bone Anabolic Response in the Calvaria Following Mild Traumatic Brain Injury is Mediated by the Cannabinoid-1 Receptor

**DOI:** 10.1038/s41598-019-51720-w

**Published:** 2019-11-07

**Authors:** Michal Eger, Miaad Bader, Dara Bree, Rivka Hadar, Alina Nemirovski, Joseph Tam, Dan Levy, Chaim G. Pick, Yankel Gabet

**Affiliations:** 1 Department of Anatomy & Anthropology, Sackler Faculty of Medicine, Tel Aviv University, Tel Aviv, Israel; 2 Departments of Anesthesia, Critical Care and Pain Medicine, Beth Israel Deaconess Medical Center and Harvard Medical School, MA, USA; 3 Obesity and Metabolism Laboratory, Institute for Drug Research, School of Pharmacy, Faculty of Medicine, The Hebrew University of Jerusalem, Jerusalem, 9112001 Israel; 4 Sagol School of Neuroscience, Tel-Aviv University, Tel-Aviv, 69978 Israel; 5 The Dr. Miriam and Sheldon G. Adelson Chair and Center for the Biology of Addictive Diseases, Tel-Aviv University, Tel-Aviv, 69978 Israel

**Keywords:** Bone, Trauma

## Abstract

Brain trauma was clinically associated with increased osteogenesis in the appendicular skeleton. We showed previously in C57BL/6J mice that mild traumatic brain injury (mTBI) transiently induced bone formation in the femur via the cannabinoid-1 (CB1) receptor. Here, we subjected ICR mice to mTBI and examined the bone response in the skull using microCT. We also measured mast cell degranulation (MCD)72 h post-injury. Finally, we measured brain and calvarial endocannabinoids levels post-mTBI. mTBI led to decreased bone porosity on the contralateral (untouched) side. This effect was apparent both in young and mature mice. Administration of rimonabant (CB1 inverse agonist) completely abrogated the effect of mTBI on calvarial porosity and significantly reduced MCD, compared with vehicle-treated controls. We also found that mTBI resulted in elevated levels of anandamide, but not 2-arachidonoylglycerol, in the contralateral calvarial bone, whereas brain levels remained unchanged. In C57BL/6J CB1 knockout mice, mTBI did not reduce porosity but in general the porosity was significantly lower than in WT controls. Our findings suggest that mTBI induces a strain-specific CB1-dependent bone anabolic response in the skull, probably mediated by anandamide, but seemingly unrelated to inflammation. The endocannabinoid system is therefore a plausible target in management of bone response following head trauma.

## Introduction

The main purpose of the skull is to protect the soft and vulnerable brain. However, brain contusion may occur when a direct impact to the skull is strong enough to produce a local inbending of the calvarial bones by applying focal pressure on the underlying brain tissue^1^. The question whether regulatory mechanisms may increase calvarial thickness following trauma to protect against future insults remains unclear. Clinical evidence exists for increased osteogenesis in patients with traumatic brain injury (TBI), which leads to heterotopic ossification and enhanced fracture healing, mainly in the appendicular skeleton^2–4^. More importantly, marked calvarial thickening was reported in infants diagnosed with shaken baby syndrome^5^. In a previous report, we showed that mild traumatic brain injury (mTBI) induces bone formation in the femur by activating the cannabinoid-1 (CB1) receptor and inhibiting norepinephrine release in bone tissue^6^. However, the increased bone formation was transient, and it did not result in a significantly increased bone volume fraction (BV/TV). We also showed that mTBI resulted in the synthesis of the main endocannabinoid, 2-arachidonoylglycerol (2-AG), by osteoblasts, thus binding the CB1 receptor in the presynaptic part of a sympathetic nerve-osteoblast contact, and inhibiting norepinephrine release. Because sympathetic norepinephrine is a tonic suppressor of osteoblast activity, mTBI alleviated this inhibition, and thus stimulated osteoblastic bone formation^7^.

Calvarial growth and homeostasis are thought to be largely controlled by the periosteal layer of the dura mater. The dura mater is also a major source of osteogenic cells during calvarial healing following an injury^8,9^. The cranial dura is populated by a large number of immune cells including mast cells (MCs) and macrophages. With regard to the calvarial bone, MCs are of particular interest, given their ability to trigger inflammation in response to bone fracture and to regulate osteoclast activity during bone remodeling following injury^10^. Upon tissue injury, activation of MCs in response to neurogenic inflammation also plays a role in mediating heterotopic ossification via the direct or indirect release of osteogenic factors^11^. We reported that mTBI in mice causes the dural MCs to become persistently activated^12^, suggesting that it plays a possible role in mediating calvarial remodeling following injury.

Notably, despite its proximity to the brain, calvarial bone innervation is part of the peripheral nervous system and includes a sympathetic nervous system component^13^. However, the cranium differs from the femur in several developmental and functional aspects. The calvaria is a flat bone that develops in a pattern of intramembranous bone formation, whereas the femur consists of endochondral bone formation. In addition, the mechanical environment and the direction of forces to which the femur is subjected differ from the calvaria. In general, the regulation of bone homeostasis and physiological adaptation differ greatly from one skeletal site to another^14,15^.

In the present study, we examined the effect of mTBI in the skull bone and investigated whether mTBI induces a bone response capable of increasing the calvarial bone density. Given the potential role of the dura mater, and particularly MCs, in cranial bone remodeling and the effect of mTBI on dural MCs, we further examined whether the effect of mTBI on cranial bone response might involve dural MCs. Our hypothesis was that the CB1 receptor mediates an adaptive osteogenic response aimed at strengthening the skull, thus optimizing protection to the brain.

## Results

### Calvarial porosity after mTBI

**Figure 1:**
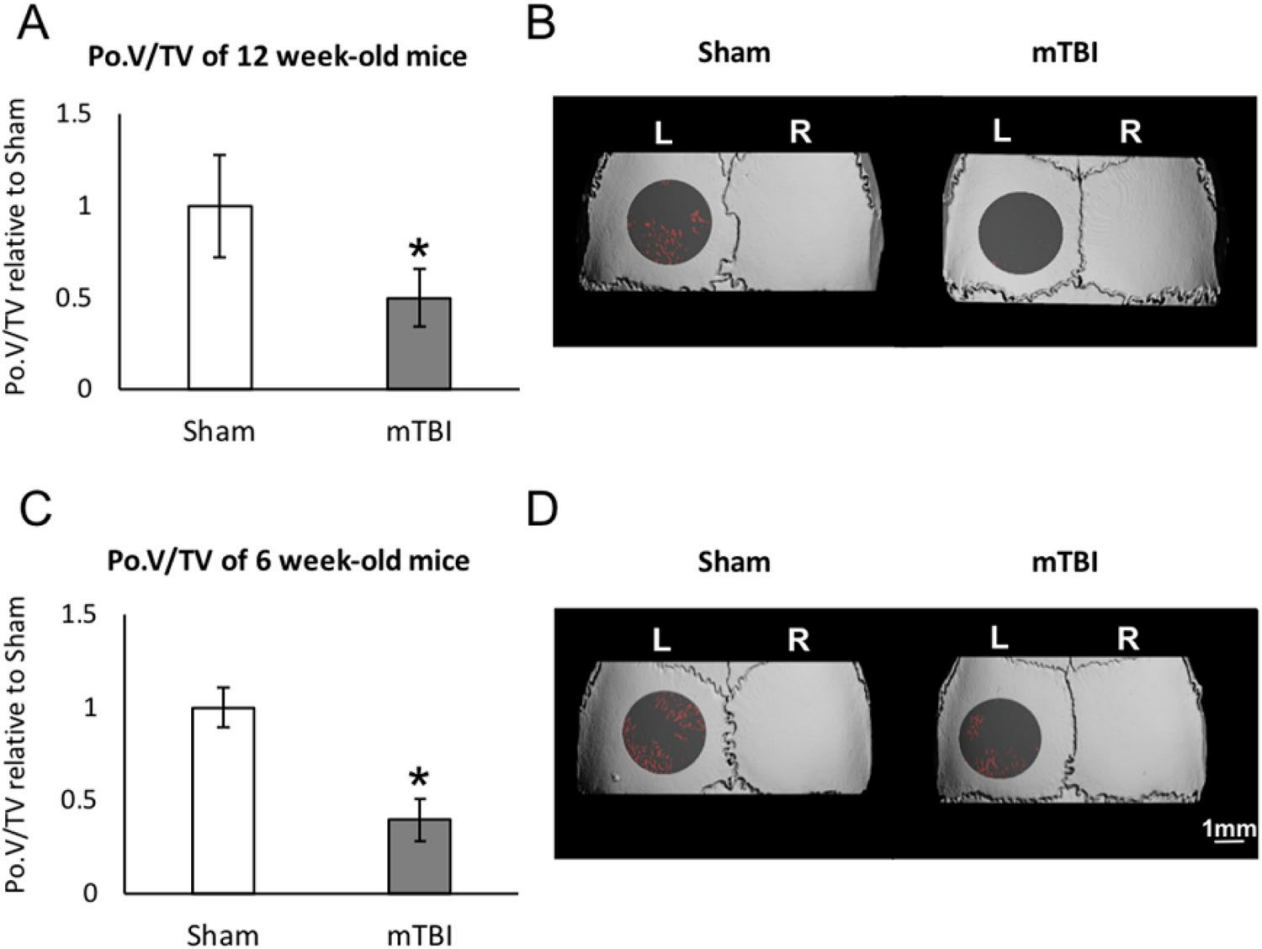
Calvarial porosity (PoV/TV) in the left parietal bone 10 days after mTBI on the right side in 12-week-old (**A,B**) and 6-week-old (**C,D**) ICR male mice. (**A**,**C**) PoV/TV mean ± SD in 9 animals per group; *p < 0.05 *vs*. sham-TBI. (**B**,**D**) Representative µCT images in the calvaria; ROI is shown as transparent gray; bone marrow spaces are shown in red.

To examine the effect of mTBI on the bone anabolic response in the calvaria, we first analyzed the effect of mTBI in skeletally mature, 12-week-old ICR mice. In general, we elected to analyze the contralateral side to preclude interference due to direct physical effects on the side subjected to the trauma. Indeed, a previous study showed that microfractures occurred on the ipsilateral side of the calvaria in most of the animals subjected to mTBI^16^. Throughout our study we found a similar trend between the right and left sides, although the ipsilateral side not always yielded statistically significant differences due to a higher variance compared to the contralateral side (data not shown). Our micro-Computed Tomography (μCT) analysis showed that mTBI on the right side leads to reduced skull porosity (i.e., increased bone mass) on the left, untouched side after 10 days (Fig. 1).

We also measured skull thickness (from the dura mater to the periosteum) and the average pore diameter. In this experiment, skull thickness was 12.6% ± 1.4 and 11.7 ± 1.1 for control vs. mTBI mice respectively, and average pore diameter was 4.1% ± 0.6 for control vs. 4.3% ± 0.6 for mTBI mice. Both parameters did not show any statistically significant differences. To determine whether the effect of mTBI on the calvarial porosity was age-dependent, we also examined young (6-week-old) mice. We found that mTBI leads to a net increase in bone volume fraction (reduced porosity) in the young mice similar to that in the mature mice (Fig. 1A,C, respectively). µCT images of the calvaria are shown in Fig. 1B,D, respectively.

**Figure 2:**
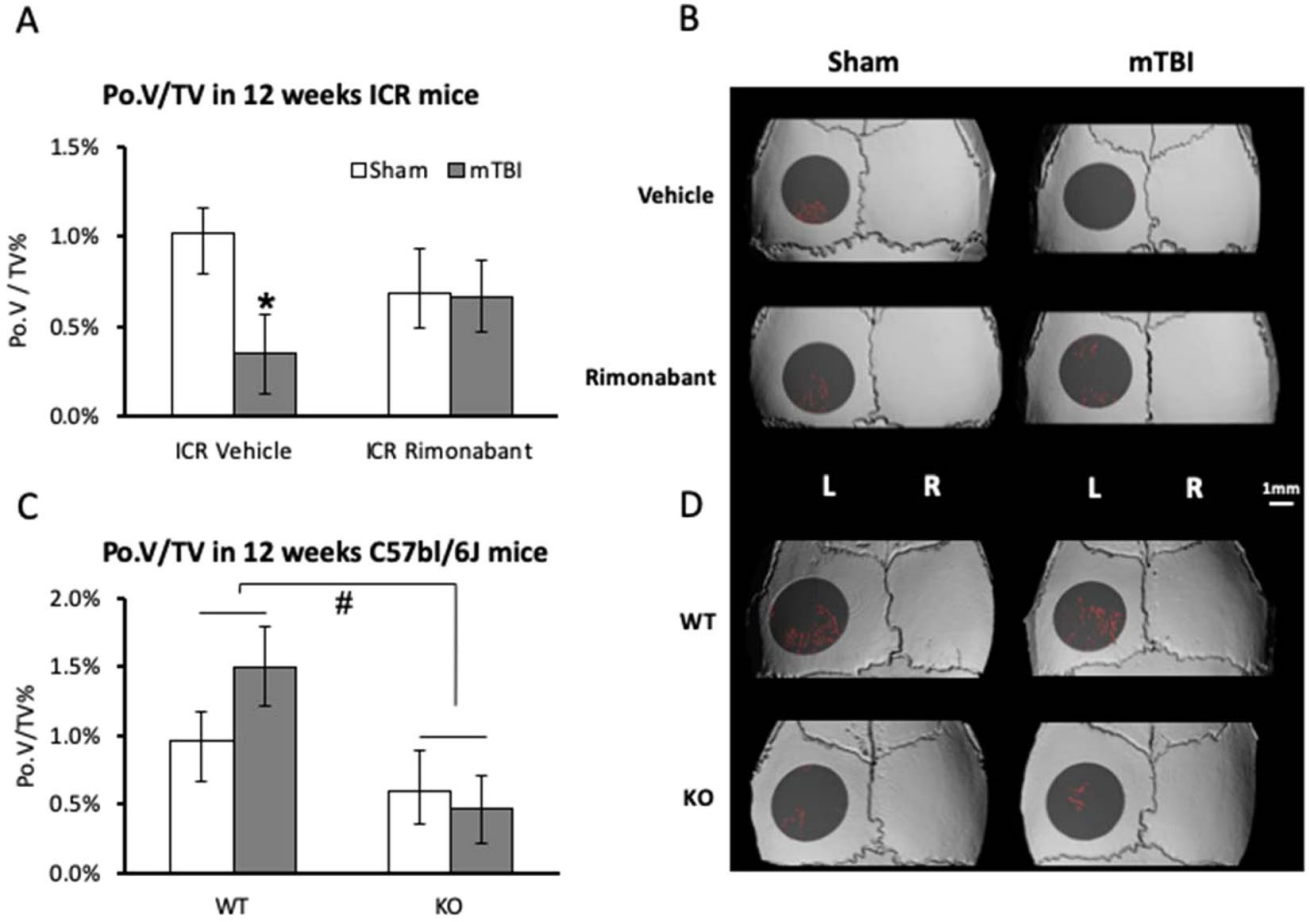
Strain-dependent role of CB1 in calvarial porosity at steady-state and following mTBI. Calvarial porosity (PoV/TV) in the left parietal bone 10 days after mTBI on the right side in 12-week old male mice. (**A**,**B**) ICR mice treated with Rimonabant or vehicle. (**C**,**D**) C57Bl/6J WT and CB1^−/−^ mice. (**A**) Graphs represent mean ± SD in 6 animals per group. *p = 0.039, vs Sham. (**C**) Graphs represent mean ± SD in 5 animals per group except n = 8 in the WT sham group. ^#^p = 0.014, KO vs WT, 2-way ANOVA) and (**B**,**D**) µCT representative images. Color coded as described in Fig. 1.

A previous study reported that mTBI induces an osteogenic response in the appendicular skeleton via CB1 receptor signaling^6^. To determine whether CB1 signaling is also involved in the calvarial response to mTBI, we systemically blocked the CB1 receptor in our 12-week old ICR mice by using the inverse agonist rimonabant, which was injected every day, starting 24 hr prior to mTBI induction. Our data clearly show that pharmacological blockade of CB1 completely abrogated the effect of mTBI on calvarial porosity (Fig. 2), suggesting that CB1 mediates the calvarial osteogenic response to mTBI.

We also used CB1 knockout versus WT mice. These mice were on a C57BL/6J background, and we subjected 12-week old male mice to the same mTBI protocol and µCT analysis. Interestingly, we observed that mTBI in this strain did not affect the skull porosity, but, independently of the mTBI, the genetic knockout of CB1 resulted in a lower calvarial Po.V/TV compared to the WT littermates.

### Degranulation of dural mast cells

**Figure 3:**
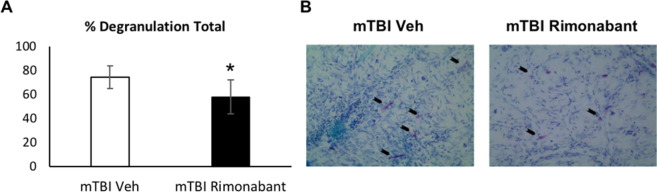
Effect of rimonabant on dural mast cell degranulation following mTBI. (**A**) Rimonabant treatment in mTBI animals resulted in a significant reduction in the dural MC degranulation level (n = 4, *p < 0.05 Rimonabant vs Veh, non-parametric Mann-Whitney). (**B**) Representative images of toluidine blue-stained dural whole-mounts showing degranulated MCs in rimonabant and vehicle-treated mTBI. Black arrows indicate degranulated mast cells.

We have previously demonstrated elevated levels of dural MC degranulation at 72 hrs following mTBI; this effect persisted up to 30 days^12^. To determine whether this inflammatory response is involved in mediating the effect of CB1 on the calvarial osteogenic response to mTBI, we examined the effect of rimonabant on mTBI-induced changes in dural MC degranulation at 72 hrs after mTBI in the ICR mice. Histological analyses revealed reduced MC degranulation levels following rimonabant treatment when compared with vehicle (p < 0.05; Fig. 3), suggesting that CB1 activation contributes to dural MC degranulation following mTBI.

### Increased endocannabinoid ‘tone’ following mTBI

**Figure 4:**
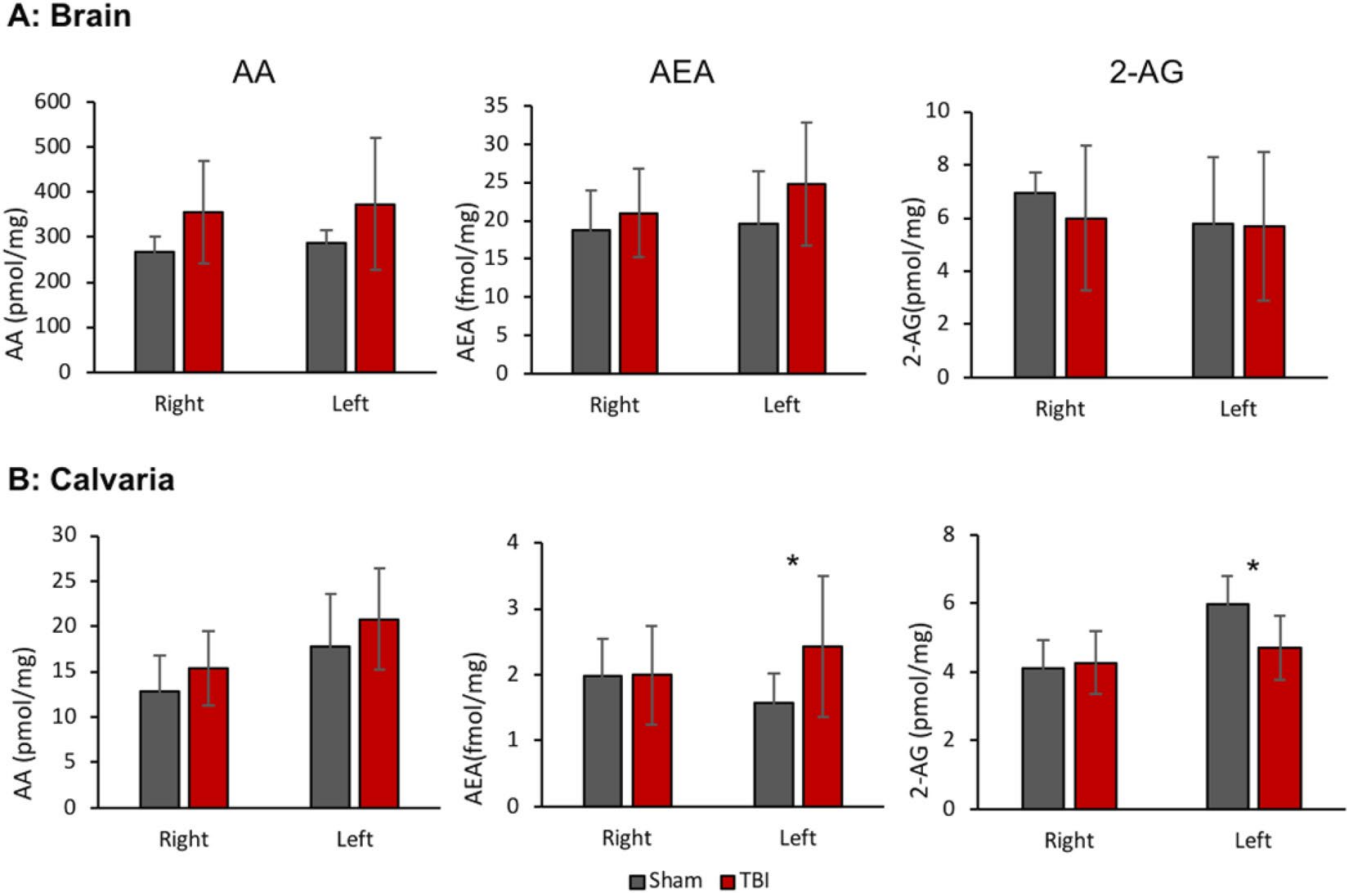
Brain (**A**) and calvarial bone (**B**) endocannabinoid levels following mTBI using LC-MS/MS. Data represent mean ± SD in 8–10 mice per group *p < 0.05 versus Sham for each side separately, using Student’s *t*-test.

The two-classical endogenous CB1 agonists are N-arachidonoylethanolamine (AEA, anandamide) and 2-arachidonoylglycerol (2-AG). These molecules are degraded to arachidonic acid (AA) and ethanolamine and AA and glycerol, respectively^17^. We therefore examined which agonist contributes to the CB1-mediated calvarial osteogenic effect of mTBI. Because we previously reported that in the appendicular skeleton the endocannabinoid response occurs within hours after the injury, we collected the brain tissue and calvarial bone 8 hrs after performing mTBI. Our analysis did not reveal any significant change in the levels of AEA, 2-AG, and AA in the brain. However, we found a significant elevation in the bone content of AEA on the left, contralateral calvaria. Bone 2-AG levels on the left side were significantly reduced. Interestingly, no significant changes in the levels of these endocannabinoids were noted in the injured side (right, Fig. 4)

## Discussion

A key finding of the current study was the osteogenic response in the calvaria contralateral to the site of the head trauma, which suggests a remote neuronal or hormonal effect rather than a direct response to the local mechanical trauma in the calvaria. We also found that this trauma-induced reduction in bone porosity occurs similarly in young and adult animals. Our data suggest that the mTBI-evoked osteogenic response in the calvaria is probably mediated via local activation of CB1 in the calvaria by elevated levels of AEA, and that it may involve a direct or indirect contribution of inflammatory mediators released by activated dural MCs.

In this study a closed head weight drop model was utilized to produce an mTBI in mice mimicking the symptoms in mTBI victims who do not show clear morphological brain deficits, but frequently suffer from lasting cognitive deficits and behavioral disturbances^18,19^. We demonstrated previously that this model causes significant cognitive impairments without any other clear neurological damage (normal neurological score), no evident brain edema, no notable damage to the blood-brain barrier and no early anatomical changes to the brain^20^. In addition, following exposure to this model the physiological, neurological, emotional, and motor function as well as pain threshold of mTBI mice (i.e. their well-being) remains largely intact^21,22^.

In a previous study, we showed that mTBI induces an osteogenic response in the femur^6^. However, this effect seemed to be short-acting, since we observed an increase in the mineral apposition rate (i.e., osteoblast activity) during the first 24 hrs following brain injury^6^, but we could not detect an increased bone mass over the following 2 weeks. Here, we observed a significant decrease in the calvarial porosity following mTBI, suggesting a more sustained osteogenic response in the skull than in appendicular long bones.

Our previous report also indicated that the mTBI-induced osteogenic response in the femur was mediated by CB1 in the neuronal end of a nerve-to-osteoblast synapse-like connection^6^. The CB1 agonist whose role was identified in that study was 2-AG. Here we confirmed that CB1 is also involved in the TBI-induced bone gain in the calvaria. The elevated levels of AEA in the bone tissue, but not in the brain, indicate the contribution of the peripheral endocannabinoid system to the mTBI-evoked bone response in the calvaria, probably via local production and secretion of AEA or by inhibiting its degradation via fatty acid amide hydrolase (FAAH).

The role of CB1 in steady-state bone remodeling is strain specific with opposite directions in C57BL/6J versus ICR/CD1 mature mice^23,24^. CB1 knockout mice on a ICR/CD1 background display a high bone mass phenotype, whereas C57BL/6J display a low bone mass phenotype in the appendicular skeleton of 12-week old mice^23,24^. Of note, CB1 knockout in the ICR/CD1 mouse strain resulted in accelerated bone loss and reduced bone density in ageing mice over the age of 12 weeks^25^. In the skull, it seems that pharmacological CB1 blockade in ICR/CD1 shows a similar skeletal outcome as genetic CB1 knockout in the C57BL/6J strain at steady-state (Fig. 2), consistent with the idea that CB1 has a bone protective role in both strains during adulthood. On the other hand, CB1 blockade by rimonabant abrogated the mTBI-induced calvarial bone gain in ICR/CD1 mice, but CB1 knockout in C57BL/6J mice did not. Our data therefore show that the calvarial osteogenic action of CB1 following mTBI in ICR/CD1 mice, is similar to that reported in the femur of C57BL/6J mice^6^, but differs from that observed in the skull of the latter strain (Fig. 2). Thus, the skeletal role of CB1 at steady-state or in response to mTBI is dependent on the genetic background, skeletal site and age.

The finding that CB1 antagonism reduced mTBI-evoked MC degranulation response points to the possibility that dural MCs may contribute, directly or indirectly, to the osteogenic response. Our earlier finding that mTBI leads to an acute degranulation of dural MC contralateral to the head injury is also in agreement with the contralateral osteogenic response in the calvaria observed herein^12^. MC degranulation, probably in response to neurogenic inflammation, has been suggested to mediate heterotopic ossification in long bones following tissue injury^11^. However, MC accumulation and degranulation have also been shown to promote bone resorption and to inhibit osteoblastic bone formation^26,27^. Further studies are required to examine the relative contribution of dural MC degranulation and the associated meningeal inflammation in mediating mTBI-induced bone formation in the skull.

Of note, the skeletal effect of mTBI reported here is conceptually distinct from the well-established stimulation of bone healing following brain trauma^28,29^. In the present study, we show a bone response in the contralateral side that is not directly affected by the weight drop. Indeed, others showed that microfractures often occur in the ipsilateral side^16^, and the effect of mTBI on bone healing is likely different from the CB1-mediated effect of mTBI in intact bone.

One clear limitation of this study is the use of experimental animals that do not always mimic clinical conditions. Further research will also be needed to fully understand the mechanism of action of CB1 as well as the contribution of mast cells is mediating the calvarial response to mTBI.

Wolff’s law indicates that bone tissue adapts to pressure. Here we show that mild brain trauma, which does not cause bone fracture, results in an osteogenic response. We speculate that the aim of this CB1-mediated adaptive mechanism is to protect the brain against future injuries by strengthening the skull. Brain trauma is induced by local inbending of the calvarial bones, resulting in a focal strain on the brain (‘coup’), and by intracranial pressure during the rebound effect (‘contre-coup’)^1^. Although unlikely to be protective against the contre-coup, we can assume that a stronger skull attenuates, at least in part, the deleterious effect of the coup. Indeed, a recent study showed that (i) skull fracture following TBI was associated with a more severe brain damage, and (ii) thicker parietal bone significantly reduced the risk of TBI-induced skull fracture^30^. Our study may also shed light on the mechanism underlying the reported skull thickening in the shaken-baby syndrome^5^.

## Methods

### Mice

Male ICR mice, and male C57BL/6J CB1 knockout and WT, aged 6 to 12 weeks, as indicated, were housed 5-per-cage under a 12-hour light/dark cycle with food (Purina Rodent Chow) and water given ad libitum. The mice were exposed to a constant temperature of 22 ± 2 °C. The Ethics Committee of the Sackler Faculty of Medicine approved the experimental protocol (M-14-037) in compliance with the guidelines for animal experimentation of the National Institutes of Health (DHEW Publication 85–23, Revised, 1995). A minimal number of mice were used, and all efforts were made to minimize potential suffering.

### Closed head mild traumatic brain injury

Mice were subjected to an mTBI procedure described in detail elsewhere^12,20,31^. Briefly, mice were anesthetized with 3% isoflurane and placed under a weight drop concussive head trauma apparatus. A 30 g weight was dropped through a guide tube from a height of 80 cm, striking the head at the temporal right side between the corner of the eye and the ear. A sponge was placed under the animals to support their head while allowing some anterior-posterior motion, without any rotational head movement during the impact. Immediately after the impact, mice were placed in their cages for recovery. Sham-TBI animals were anaesthetized, but not subjected to weight-drop. This procedure has been validated repeatedly in our group, as it creates a highly reproducible mTBI model^20–22^. When indicated, rimonabant (10 mg/kg, Sigma), or vehicle (DMSO:Tween80:saline at a concentration of 5:1:39) was injected intraperitoneally to the mice once a day starting 24 hr before the mTBI and until euthanasia. Tissues were harvested either 8 hr post-mTBI for molecular response, 72 hr post-mTBI for studying the dural MC response, or after 10 days to assess the osteogenic response using micro-computed tomography (μCT).

### Micro-Computed Tomography (μCT)

After a follow-up period of 10 days, the mice were anesthetized with Ketamine (100 mg/kg) and xylazine (10 mg/kg, i.p.) and perfused transcardially with phosphate-buffered saline and then with 4% paraformaldehyde (PFA) in 0.1 M phosphate buffer, pH 7.4. The skulls were removed, fixed for 24 hr in 4% phosphate-buffered formalin (PBF), followed by 24 hr in 1% PBF, and then stored in 70% ethanol. All specimens were scanned and analyzed using a μCT50 system (Scanco Medical AG, Switzerland). Scans were performed at an isotropic resolution of 10 μm, with 90 kVp energy, at 88 μA intensity, and with 1000 projections at a 1000 msec integration time. The region of interest (ROI) was defined as two 3.0 mm circles in the center of the parietal bones.

The mineralized tissues were differentially segmented using a global thresholding procedure^32^. A custom-made algorithm, based on Image-Processing Language (IPL, Scanco Medical), was developed to isolate the diploe marrow spaces inside the ROIs. The total volume of marrow spaces (porosity volume, Po.V, μm^3^), and the total volume of the calvarial bone in the ROIs (TV, μm3) were measured at the 3D level to calculate the porosity volume fraction (Po.V/TV, %). This parameter is the inverse of the more commonly reported bone volume fraction (BV/TV). Results are normalized to the sham-TBI group (Po.V/TV = 2.9% ± 0.8 in a representative experiment using 12 wk old mice).

### Measurement of tissue endocannabinoid levels

Eight hours after mTBI, animals were euthanized by a cervical dislocation following isoflurane sedation. The brain and calvarial tissues were separated and snap-freezed in liquid nitrogen. Tissue levels of the endocannabinoids (eCBs) AEA, and 2-AG, as well as their degrading molecule arachidonic acid (AA) were measured in the brain and parietal bone, using the stable isotope dilution LC-MS/MS method as described previously^33^. Briefly, bone and brain samples were homogenized in 0.5 mL of ice-cold methanol/Tris buffer (50 mM, pH 8.0), 1:1, containing [^2^H4]AEA as an internal standard. Homogenates were then extracted with CHCl_3_:MeOH (2:1, vol/vol), and washed three times with ice-cold CHCl_3_, dried under nitrogen flow, and reconstituted with MeOH. Then, total proteins were precipitated. LC-MS/MS analyses were conducted on an AB Sciex (Framingham, MA, USA) Triple Quad 5500 mass spectrometer coupled with a Shimadzu (Kyoto, Japan) UHPLC System. Liquid chromatographic separation was carried out using a Kinetex (Phenomenex) column (C18, 2.6 µm particle size, 100 × 2.1 mm). eCBs were detected in a positive ion mode under ESI and MRM conditions. The molecular ions and fragments for each compound were m/z 348.3 → 62.1 (quantifier) and 91.1 (qualifier) for AEA, m/z 379.3 → 91.1 (quantifier) and 287.3 (qualifier) for 2-AG, m/z 305.2 → 91.1 (quantifier) and 77.1 (qualifier) for AA, and m/z 352.3 → 66.1 (quantifier) and 91.1 (qualifier) for [^2^H4]AEA. The levels of AEA, 2-AG, and AA in the samples were measured against standard curves.

### Histological analysis of dural mast cell degranulation

Animals intended for cellular analysis in the dura were anaesthetized and perfused as described above. The skulls were removed and post-fixed overnight in the same fixative solution, and then transferred to 1% PFA. The dura was then detached from the skull bone and mounted on a glass slide^12^. To quantify the MC degranulation level, fixed dural whole-mount tissues were stained with toluidine blue (0.1% in 2.5 pH), which binds to glycosaminoglycans in connective tissue MC granules. MC density and degranulation levels were determined using bright-field illumination under a ×400 magnification (Nikon, Eclipse Ci, Japan). Because MC density and degranulation levels were uniform across the dura on each side, MC counts and degranulation levels on each side were averaged, based on 10 different randomly chosen visual fields. Dural MCs were considered degranulated if there was an extensive dispersion of more than 15 extruded granules localized near the cell, or when there was an extensive loss of granule staining, giving the cell a ‘ghostly’ appearance^34,35^. MC counts and degranulation levels were conducted in a blinded fashion.

### Statistical analyses

Values are expressed as mean ± SD. All statistical analyses were performed using PRISM software v.7 (GraphPad Software, La Jolla, CA). The MC data were analyzed using the non-parametric Mann Whitney U test. To compare sham-mTBI to mTBI mice in all other experiments (n > 6 and normal distribution), we used a two-tailed Student’s *t*-test. When comparing the effect of *CB1-*knockout *vs*. WT and TBI *vs*. Sham-TBI in these mice, we used a 2-Way ANOVA test. The difference between groups was defined as *p* < 0.05.

## Data Availability

I confirm that my article contains a Data Availability Statement. The authors will make raw data, processed data, software, algorithms, protocols, details methods and materials available upon request.
