# Author Correction: Bone Anabolic Response in the Calvaria Following Mild Traumatic Brain Injury is Mediated by the Cannabinoid-1 Receptor

**DOI:** 10.1038/s41598-020-73944-x

**Published:** 2020-10-09

**Authors:** Michal Eger, Miaad Bader, Dara Bree, Rivka Hadar, Alina Nemirovski, Joseph Tam, Dan Levy, Chaim G. Pick, Yankel Gabet

**Affiliations:** 1 Department of Anatomy & Anthropology, Sackler Faculty of Medicine, Tel Aviv University, Tel Aviv, Israel; 2 Departments of Anesthesia, Critical Care and Pain Medicine, Beth Israel Deaconess Medical Center and Harvard Medical School, Boston, MA USA; 3 Obesity and Metabolism Laboratory, Institute for Drug Research, School of Pharmacy, Faculty of Medicine, The Hebrew University of Jerusalem, 9112001 Jerusalem, Israel; 4 Sagol School of Neuroscience, Tel-Aviv University, 69978 Tel-Aviv, Israel; 5 The Dr. Miriam and Sheldon G. Adelson Chair and Center for the Biology of Addictive Diseases, Tel-Aviv University, 69978 Tel-Aviv, Israel

Correction to: *Scientific Reports* 10.1038/s41598-019-51720-w, published online 7 November 2019.

The original version of this Article contained a typographical error in the spelling of the author Alina Nemirovski, which was incorrectly given as Alina Nemerovski. This has now been corrected in the PDF and HTML versions of the Article.

